# Management of Essential Blepharospasm

**Published:** 2017-06-19

**Authors:** Eric Clayman, Kathryn S. King, Michael A. Harrington

**Affiliations:** Division of Plastic Surgery, Department of Surgery, University of South Florida Morsani College of Medicine, Tampa

**Keywords:** essential blepharospasm, dyskinesia, dystonia, blepharoplasty, direct brow lift

## DESCRIPTION

A 63-year-old man presented with a history of intermittent difficulty opening eyes with need for excessive ocular muscle use diagnosed with essential blepharospasm, bilateral brow ptosis, and bilateral dermatochalasis. He underwent bilateral direct brow lift and upper eyelid blepharoplasty with orbicularis resection.

## QUESTIONS

1. **What is essential blepharospasm?**
2. **What causes essential blepharospasm?**
3. **What are the currently accepted management options for essential blepharospasm?**
4. **What are the potential future management options for essential blepharospasm?**

## DISCUSSION

Benign essential blepharospasm (BEB) is a craniofacial dyskinesia characterized by repeated, involuntary contractions of the orbicularis oculi, procerus, and corrugator muscles.^1^^,^^2^ These spasms cause involuntary blinking that can become severe and lead to functional blindness.^2^ BEB has a prevalence of 5 to 13 per 100,000 individuals, is almost 3 times more common in females, and has a median age of diagnosis at 53 years of age.^3^^,^^4^

Normal blinking involves alternating activity of eyelid protractors (orbicularis oculi, procerus, and corrugator) and retractors (levator palpebra and frontalis). In BEB, the mutual inhibition of these muscle groups is lost, resulting in spasm. The exact pathophysiology behind development of BEB is unknown but is thought to be multifactorial, with a likely genetic component and environmental trigger. It has been suggested that BEB may be partly due to abnormalities in the basal ganglia and dopaminergic system involving a subclinical loss of striatal dopamine. This loss of dopamine creates a vulnerability of the trigeminal blink circuit to excitation by an environmental insult or external trigger. There is also a progressive loss of dopamine in the substantia nigra that occurs with aging. Therefore, there is an increased excitability of the trigeminal blink reflex with age, and this association correlates with the fact that BEB is typically diagnosed at a later age.^5^

The most effective treatment options for BEB at this time are chemodenervation and surgical intervention. Chemodenervation with Botox injections, Food and Drug Administration approved for BEB in 1989, provides symptomatic relief in approximately 73% of patients. Symptom resolution from botulinum toxin is transient and requires repeated injections every few months for the remainder of the patient's life. Many patients develop more severe disease and/or clinical resistance to botulinum toxin. The preferred treatment of BEB refractory to botulinum toxin is a surgical upper eyelid myectomy/blepharoplasty. This surgical approach was the original treatment option for BEB prior to the commercial availability of botulinum toxin and involves excision of the pretarsal and preseptal orbital orbicularis oculi muscles. Upper eyelid myectomy decreases the morbidity, frequency of botulinum toxin treatment, and long-term expense associated with BEB management.^1^

There are a minority of patients who do not get complete symptomatic relief following myectomy and may benefit from alternative therapies.^5^ Karapantzou et al^6^ proposed a minimally invasive frontalis suspension surgery for patients unresponsive to botulinum toxin injections. This technique involves subcutaneous insertion of polytetrafluoroethylene sutures from the edge of the upper eyelid to the caudal portion of the frontalis muscle. This technique provided a mean improvement of 70%; however, all patients required additional botulinum toxin injections for optimal outcomes.^6^ Repetitive transcranial magnetic stimulation (rTMS) is another potential novel therapeutic option for patients with BEB. Low-frequency rTMS over the anterior cingulate gyrus was shown to be safe and to improve clinical symptoms of BEB.^7^ Methylphenidate has emerged as a potential therapeutic pharmacologic intervention in the management of BEB. Price et al^5^ found that there was a significant decrease in surface electromyographic voltages over the lower eyelid pretarsal and preseptal orbicularis oculi muscles as well as an improvement in Functional Disability scores after administration of methylphenidate.^5^ These potential future management options may provide opportunities for complementary treatment modalities for minimization of symptoms and improvement in quality of life for patients with BEB.

The patient presented to our office as a referral from the neuro-ophthalmologist for surgical treatment of his blepharospasm after failed pharmacologic treatment. Given the presence of brow ptosis and dermatochalasis on examination, the decision was made to proceed with surgical treatment with brow lift, blepharoplasty, and myectomy for functional visual improvement.

## Figures and Tables

**Figure 1:**
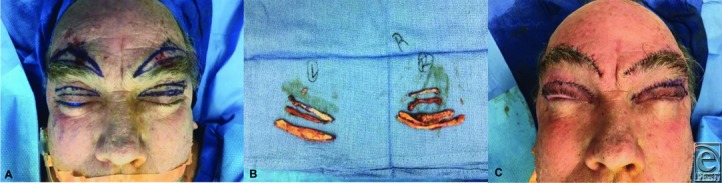
(a) Preoperative markings for bilateral brow lift and blepharoplasty. (b) Skin and muscle resection. (c) Immediate postoperative result.
